# Prevalence of Schistosoma Haematobium Measured by a Mobile Health System in an Unexplored Endemic Region in the Subprefecture of Torrock, Chad

**DOI:** 10.2196/13359

**Published:** 2019-06-18

**Authors:** Didier Lalaye, Mirjam E de Bruijn, Tom PVM de Jong

**Affiliations:** 1 Julius Global Health Center University Medical Center Utrecht Utrecht Netherlands; 2 African Studies Centre Leiden University Leiden Netherlands; 3 Department of Pediatric Urology University Children’s Hospitals University Medical Center Utrecht and Amsterdam University Medical Center Utrecht Netherlands

**Keywords:** Schistosoma haematobium, prevalence, Chad, neglected tropical diseases, mobile health

## Abstract

**Background:**

*Schistosoma haematobium* is a parasitic digenetic trematode responsible for schistosomiasis (also known as bilharzia). The disease is caused by penetration of the skin by the parasite, spread by intermediate host molluscs in stagnant waters, and can be treated by administration of praziquantel. Schistosomiasis is considered to be an important but neglected tropical disease.

**Objective:**

The aim of this pilot study was to investigate the prevalence of schistosomiasis in the subprefecture of Torrock, an endemic area in Chad where no earlier investigation had been conducted and no distribution system for pharmacotherapy has ever existed.

**Methods:**

This study examined 1875 children aged 1 to 14 years over a period of 1 year. After centrifugation, urine examination was performed by a direct microscopic investigation for eggs. The investigation was conducted with a mobile health (mHealth) approach, using short message service (SMS) for communication among parents, local health workers, a pharmacist, and a medical doctor. An initial awareness campaign requested parents to have their children examined for schistosomiasis. Urine was then collected at home by the parents following the SMS request. Urine results that proved positive were sent to a medical doctor by SMS, who in turn ordered a pharmacist by SMS to distribute praziquantel to the infected children.

**Results:**

Direct microscopic examination of urine found 467 positive cases (24.9% of the total sample). Of all male and female samples, 341 (34%) and 127 (14.4%) samples were positive, respectively. The infection rate was equally distributed over age groups. The newly developed mHealth system had a limited level of participation (8%) from an estimated total of 25,000 children in the target group.

**Conclusions:**

The prevalence of schistosomiasis in children in the subprefecture of Torrock is moderately high. Efforts will be required to enhance the awareness of parents and to reach a larger percentage of the population. Systematic governmental measures should be put in place as soon as possible to increase awareness in the area and to diagnose and treat cases of schistosomiasis.

## Introduction

Schistosomiasis of the urogenital tract is a parasitic disease caused by *Schistosoma haematobium* (Bilharz), a flatworm of the class trematode of the genus *Schistosoma*, whose habitat is the venous circulatory system of the urinary tract and internal genitalia. The disease is spread by snails that live in stagnant infected water and act as intermediate hosts for the trematode. The snails expel Cercaria, a free swimming larval stage, in the water, which are able to penetrate the skin of people or cattle that are in the water. Once in the body, the Cercaria migrate to the blood vessels surrounding the urogenital system and develop into adult worms. The female part of a pair of worms produces eggs that penetrate into the bladder producing symptoms of lower urinary tract disease and changes of the bladder wall that may lead to malignancies of the bladder at a later age. Clinical signs may vary from hematuria with few extra complaints to severe forms of irritative bladder complaints and obstruction of the upper urinary tract caused by fibrosis of ureters. The eggs are excreted with the urine into the water and enter the snail to finish the cycle. To diagnose, the disease direct detection of eggs under a microscope in a centrifuged urine sample and detection of hematuria by urine test strips are the most commonly used tests [1].

Schistosomiasis often results in severe physical, social, and economic disabilities [2]. Schistosomiasis is a major public health problem in endemic tropical and subtropical countries. After malaria, it occupies the first place in importance in public health with regard to its prevalence [3]. In contrast to malaria, which affects people without discrimination of social position or income, schistosomiasis primarily affects poor people in remote areas [1]. This is a consequence of inadequate water, Sanitation, and hygiene conditions, based on poor infrastructure and habits in agriculture and livestock farming. This may be one of the important reasons for schistosomiasis being considered a neglected tropical disease.

In Africa, schistosomiasis affects more than 200 million people in rural or periurban areas, of whom 120 million show symptoms of the disease. For 20 million of those infected, this may have severe consequences because of increased mortality risk. In many areas, many adults and a high proportion of children under the age of 14 are infected. It is estimated that 650 million people worldwide live in endemic areas [4].

Urogenital schistosomiasis occurs in tropical and subtropical areas of the world where climatic, ecological, and socioeconomic conditions favor the spread of the disease. The risk of infection is high particularly in flood and irrigation areas [5].

In most endemic areas, the disease is diagnosed only incidentally when the patient consults a health worker for other reasons. As a result, many cases go unnoticed and the disease is rarely recognized as a cause of death in Sub-Saharan Africa, although there are reports of 280,000 deaths per year [6]. Treatment of schistosomiasis is relatively simple, by the administration of the antihelmintic medication praziquantel [7].

A survey in 2010 by the Koyom District Hospital in Chad on the prevalence of helminthiasis and schistosomiasis among rural students in Chad found a prevalence of 25% of urinary schistosomiasis [8]. In many countries, mass drug administration programs exist where the population at risk is treated routinely with praziquantel. In Chad, no reliable data exist on the prevalence of the disease, given the absence of a national program for the control of schistosomiasis and also because the organization of the health care system does not provide diagnostics to prove this pathology—thus hindering the pathway to treatment. The World Health Organization provides data on the numbers of people at risk for schistosomiasis in Chad [9].

Over 2016, more than 3 million people were considered to be in need for yearly drug administration, none have been reported receiving medication.

Our pilot study aimed to determine the prevalence of schistosomiasis in the population of Torrock and to test the effectiveness of a mobile health (mHealth) system installed locally. This followed a pilot study conducted in 2014 at the Torrock health center, which revealed 157 positive cases (26%) from a total of 611 children aged 1 to 14 years.

## Methods

The study was conducted between March 2015 and March 2016, based on a sampling and treatment system and controlled by short message service (SMS) on mobile telephones.

### Study Zone: Mayo-Kebbi Ouest

**Figure 1:**
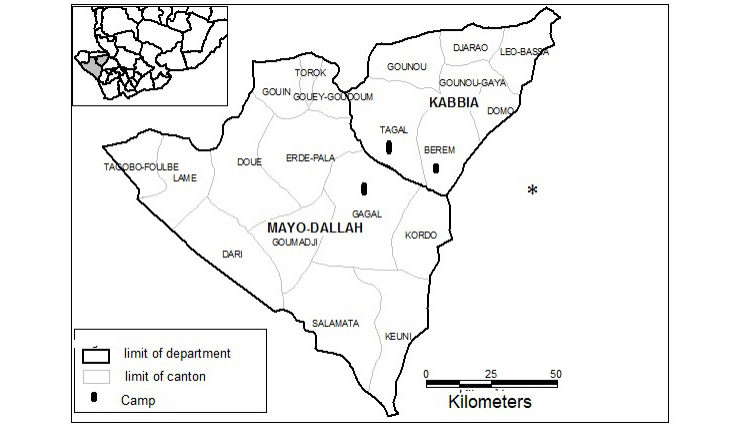
Map of Mayo Kebbi Ouest.

In Chad, the subprefecture of Torrock is in the department of Mayo-Kebbi West, located 70 km north of Pala, the capital of the province (see Figure 1). According to the last census (2009), the population is 50,000 inhabitants, with an estimated percentage of 50.6% for children between 1 and 15 years [10]. Torrock is composed of 3 cantons and has a total of 9 health centers, none of which had laboratory facilities before the start of the study. Running alongside the village of Torrock, where the sample population was chosen, there is a river called El Madorbob, the main source of molluscs that act as intermediate hosts of schistosomiasis.

No earlier studies, except for a small pilot study carried out by the first author, had been conducted in this area, and no program for distribution of medication had ever existed there.

The population had earlier been informed of the possibility to have children tested for schistosomiasis by urine sampling at home, whether they showed symptoms or not. This information was spread via churches, mosques, markets, schools, mouth to mouth, and FM radio stations serving the region both in the local language Moundang and in French. Information was given by the local announcer by megaphone, by imams in mosques, and by priests in churches. Information contained specific items on most aspects of urinary schistosomiasis.

Following this awareness campaign in the first half of March 2015, an SMS relay system was set up, which allowed children to have their urine examined at laboratories in 3 village health centers: Torrock, Gouin, and Goy-Goudoum, all in the subprefecture of Torrock. These laboratories perform limited examinations. Urine analysis for *Schistosoma haematobium* was unavailable before the study. During the study, urine analysis for *Schistosoma haematobium* was provided free of charge for the study group. After positive testing, the correct dosage of praziquantel for a child was sent to his or her home after SMS contact with a medical doctor, who prescribed the medication by SMS to the regional pharmacist.

**Figure 2:**
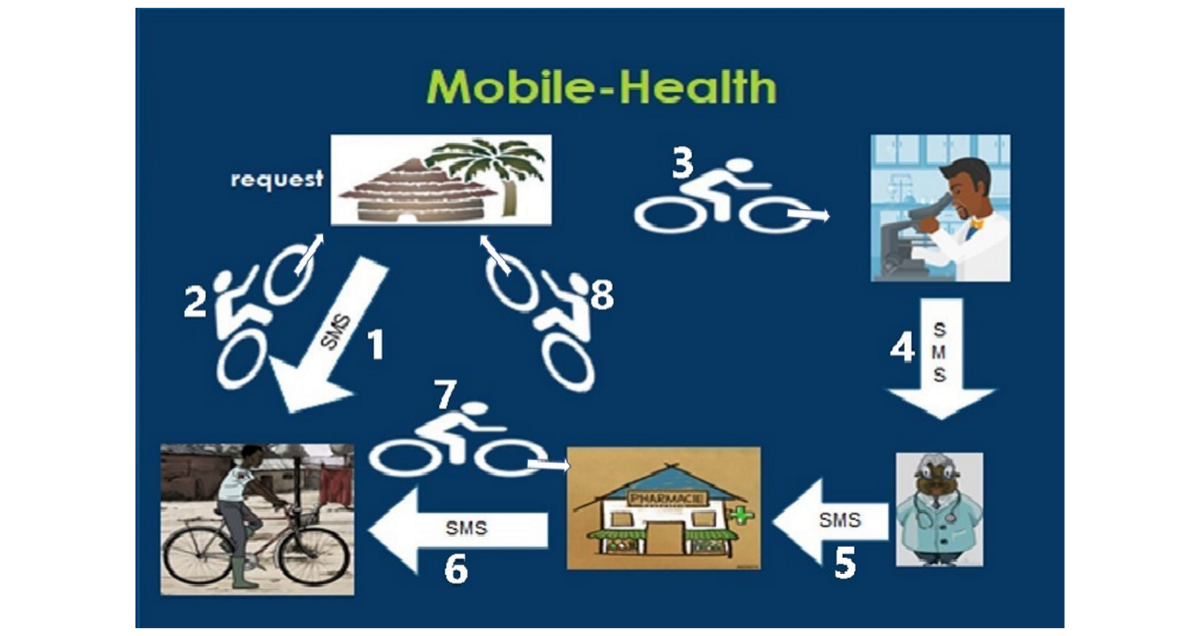
The mobile health flow diagram.

The chain for diagnostics and treatment (Figure 2 provides a graphic example of the SMS health system. The numbers refer to the different steps in the procedure) starts with a representative in the village with a mobile phone, these were 4 males, appointed by the chiefs of the villages. They are responsible for sending test request messages to the liaison officer from parents who are unable to do so themselves and were paid for each patient by the project. They link the parents to the diagnostic and therapeutic system. The liaison officer receives the message from a parent or a representative, bicycles to the area and takes the urine sample from the child’s house to the health center for an analysis. The liaison officer also weighs the child, records the child’s name, and creates a personal code. The officer is also responsible for delivery of the praziquantel in cases of a positive diagnosis. The liaison officer was appointed by the health centers and provided with a bicycle and was paid a small amount of money for each patient.

The laboratory worker in the health center performs analyses of the urine collected by the liaison officer. He informs the physician of the positive cases of schistosomiasis by noting name, code, and weight of the child. They did not receive extra payment for the project other than their regular salary.

The physician receives the urine results from the laboratory via internet or SMS, calculates the dosage of the drug according to the weight of the child, and issues orders to the pharmacist.

The pharmacist is responsible for delivering medication with the patient’s name and personal code on the package. The pharmacist sends an SMS message to the liaison officer, who delivers the medication to the families. For this study, 1 health worker trained for doing microscopy of urine samples, 1 liaison officer, 1 pharmacist, and 1 doctor were sufficient. Praziquantel was provided for free by the government district hospital.

### Laboratory Test

Laboratory equipment consisted of dry tubes for urine collection, a mechanical centrifuge operated by hand, and an optical microscope (Olympus GX 201). The urine samples were collected between 9 am and 1 pm, because this is the period that maximal egg load in the urine can be expected [11].The urine was collected in dry tubes and transported to the corresponding health center for analysis. The examination technique consisted of centrifugation for 10 min. The supernatant was discarded, and a drop of the centrifugate was examined under the microscope between glass slides. The microscopic reading of the samples was performed at magnification 100, and eggs were confirmed at magnification 400. All samples containing eggs measuring 65 to 145 μm, with a thick, pink, transparent shell with a terminal spur, were considered positive. The number of eggs of *Schistosoma haematobium* was not quantified, so there was no assessment of the intensity of the infection. Reason to do so was that the laboratory workers were not trained in counting eggs per field.

### Inclusion Criterion

All children aged 1 to 14 years whose parents had made a request for urine testing by SMS and whose children visited the health center of their own accord or as a result of parental request.

### Exclusion Criteria

Children whose parents did not make an SMS request, children under 1 year of age, and individuals 15 years of age and older.

### Ethical Considerations

Regional health authority in the province of Mayo Kebbi Ouest granted written permission for this study. Before commencing the study and before the start of the campaign to raise awareness, an application for authorization was sent to the subprefect of Torrock and the various administrative, religious, and educational authorities of the village. An authorization was granted by the health delegate of Mayo-Dallah for the establishment of 3 laboratories in the region for the analysis of urine. An institutional ethics committee did not exist in Chad at the start of the study.

Oral consent was given by the parents after explanation of the study’s aim and procedure. Written consent was impossible because the vast majority of the population is illiterate. Data were collected anonymously.

## Results

Out of an estimated number of approximately 25,000 children in the target group, 1875 requests for urine investigation were received from parents, which reflects 8% of the target population.

**Figure 3:**
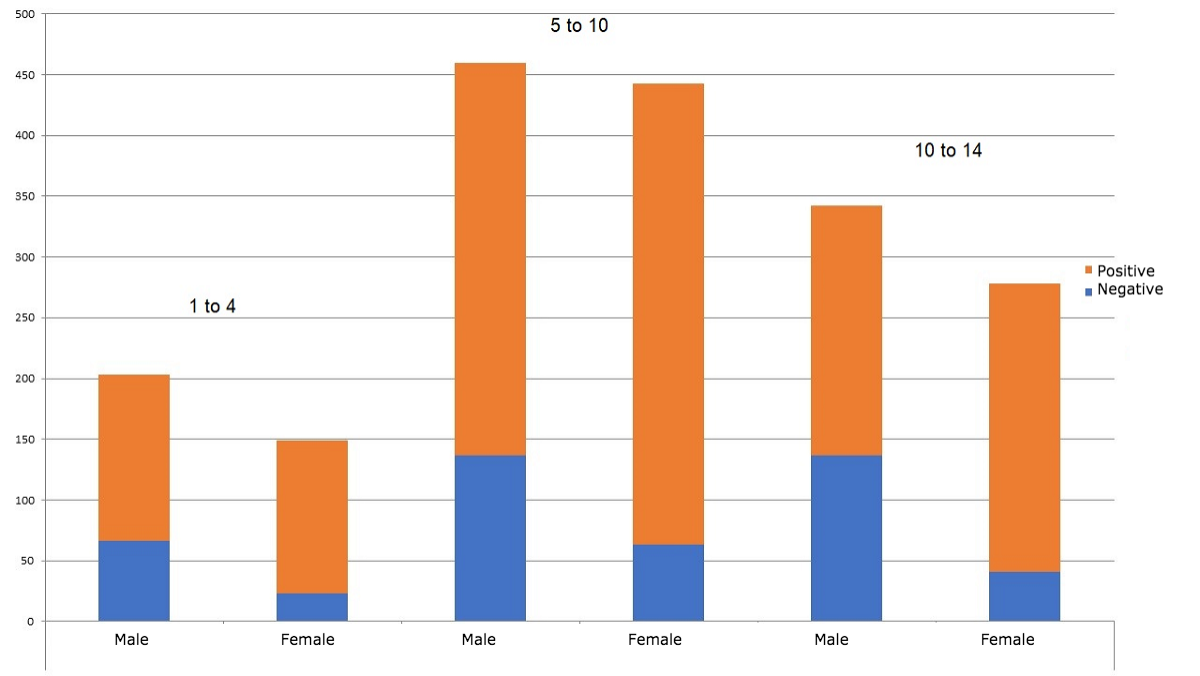
Distribution of the infected population by age group and gender.

In 1 year, a total of 1875 home-based samples were investigated. Of 1875 urine samples, 467 cases proved positive (24.9%). The results are summarized in Table 1 and Figure 3, showing that prevalence was higher in males than in females.

Total rate of infection was approximately 25% in each age group, with an infection rate of 34% and 14% in males and females, respectively. Specified for age, males, and females 1 to 4 years old combined gave 25% (88/353) positive, 32% (64/202) positive with males, and 16% (24/151) positive with females. Males and females combined 5 to 10 years old gave 25% (200/803) positive, 30% (138/460) positive with males, and 18% (62/343) positive with females. Combined 11 to 14 years old gave 25% (179/718) positive, 33% (139/422) positive with males, and 14% (40/296) positive with females.

**Table 1:** Distribution of the presence of *Schistosoma haematobium* in the study population by gender.

Gender, n (%)	Positive, n (%)	Negative, n (%)	Total, n (%)
Male	341 (34)	664 (66)	1005 (53.6)
Female	126 (14.6)	743 (85.4)	870 (46.4)
Total	467 (24.9)	1408 (75.1)	1875 (100)

## Discussion

### Principal Findings

This study revealed a prevalence of 24.9% of *Schistosoma haematobium* infection in our cohort aged 1 to 14 years (see Figure 3). This result corresponds to a study in a neighboring province, Koyom, which had an infection rate of 27.4% [8]. The 2 provinces have several points in common (rice fields and stagnant streams). This rate is lower than 2 studies in Nigeria, which had a prevalence of 40% and 47% [12,13]. The differences can be explained by the different landscape and the fact that the research in Nigeria was conducted in flood zones, where the activities of the population are based primarily on fishing and rice cultivation.

Of the total prevalence of 24.9%, the gender distribution is 73% males and 27% females. This distribution between genders is similar to that found by Dankoni and Tchuenté in northern Cameroon in 2014 [14]. They found a prevalence of 2.5% in male subjects versus 1% in females. The higher prevalence in males can be explained by the fact that boys are more active in stagnant water. It is acceptable for boys to be naked in the water up until puberty, whereas girls have to cover themselves at all ages. As a consequence, boys are more exposed to the parasite.

Surprisingly, no differences in prevalence are seen in different age groups. One would expect that a lower infection rate would be present at an early age, when children are under parental control and their only contact with stagnant water is in the company of their parents. The group aged 5 to 10 years has more freedom and also takes care of livestock, whereas the group aged 11 to 14 years should have less exposure as they are at school. This is in contrast with studies carried out in 2014 by Dankoni and Tchuenté in Cameroon [14] and by Senghor et al in Senegal [15], who found a peak, respectively, in the group aged 7 to 10 years and 7 to 12 years. Although the sample size of the group 1 to 4 years old is relatively small compared with the other age groups, it is possible that parents have been biased by clinical signs in their child. We have no data to support this.

This study in Chad is the first study, after our earlier survey, into *Schistosoma haematobium* in this large endemic area. A limitation of this study was the fact that selection was made by requesting SMS contact from parents. Although all parents in the area have access to a local representative that can send an SMS, only 8% chose to do so. When asked specifically after the information campaign, 60 out of 100 parents claimed they did not receive or did not understand the information well enough. Another limitation of the study is that only microscopy of the urine samples has been used to detect infection. This may underestimate the prevalence because of a lower detection rate compared with dipstick use for hematuria. Finally, the sample size is relatively small by the use of a bicycle. Future studies will investigate methods for optimization of information dissemination to the local people, the results of treatment, the rate of reinfection, and the difference in infection prevalence in areas with or without mHealth systems. The mHealth system proved to be cheap, quick and effective, useful in such remote contexts, and easy to operate.

### Conclusions

This study, based on the use of an mHealth care system, investigated a total of 1875 home samples of urine and found a prevalence of 24.9% of children aged 1 to 14 years infected with schistosomiasis. This demonstrates the extent of this pathology in the study area, and we hope that this model of *mHealth*, as the first phase of management of schistosomiasis in the region, will prove to be effective. Follow-up studies are currently underway to demonstrate the efficiency of the system.

## Abbreviations

mHealth: mobile health
SMS: short message service
